# Bipolymeric Pectin Millibeads Doped with Functional Polymers as Matrices for the Controlled and Targeted Release of Mesalazine

**DOI:** 10.3390/molecules25235711

**Published:** 2020-12-03

**Authors:** Dorota Wójcik-Pastuszka, Aleksandra Potempa, Witold Musiał

**Affiliations:** Department of Physical Chemistry and Biophysics, Faculty of Pharmacy, Wroclaw Medical University, ul. Borowska 211A, 55-556 Wroclaw, Poland; dorota.wojcik-pastuszka@umed.wroc.pl (D.W.-P.); aleksandra.potempa@o2.pl (A.P.)

**Keywords:** mesalazine, drug release, kinetics, colon targeted drug delivery system

## Abstract

Targeted drug delivery systems are a very convenient method of treating inflammatory bowel disease. The properties of pectin make this biopolymer a suitable drug carrier. These properties allow pectin to overcome the diverse environment of the digestive tract and deliver the drug to the large intestine. This investigation proposed bipolymeric formulations consisting of the natural polymer pectin and a synthetic polymer containing the drug 5-aminosalicylic acid. Pectin beads were prepared via ionotropic gelation involving the interaction between the hydrophilic gel and calcium ions. The obtained formulations consisted of natural polymer, 5-aminosalicylic acid (5-ASA) and one of the synthetic polymers, such as polyacrylic acid, polyvinylpyrrolidone, polyethylene glycol or aristoflex. The release of the drug was carried out employing a basket apparatus (USP 1). The acceptor fluid was pH = 7.4 buffer with added enzyme pectinase to reflect the colon environment. The amount of the released drug was determined using UV-Vis spectrophotometry at a wavelength of λ = 330 nm. The kinetics of the drug dissolution revealed that none of the employed models was appropriate to describe the release process. A kinetic analysis of the release profile during two release stages was carried out. The fastest drug release occurred during the first stage from a formulation containing pectin and polyethylene glycol. However, according to the applied kinetic models, the dissolution of 5-ASA was rather high in the formulation without the synthetic polymer during the second stage. Depending on the formulation, 68–77% of 5-ASA was released in an 8-hour time period. The FTIR and DSC results showed that there was no interaction between the drug and the polymers, but interactions between pectin and synthetic polymers were found.

## 1. Introduction

Biopolymers such as pectin have been employed in various industrial fields including the pharmaceutical industry. The compounds have been proposed as drug carriers, particularly for targeted drug delivery. Biopolymers can be used as hydrogels, films and nanoparticles very easily. Pectin can overcome various environmental conditions, especially the low pH values in the stomach, and is digested by the microflora in the colon. Due to the properties of pectin, oral administration is the preferred method of its application [1]. Pectin formulations containing a drug can be used for colon-targeted drug delivery. The carrier is capable of protecting the drug, allowing it to reach the colon. Drug release and absorption do not take place in the stomach or in the small intestine [2]. The advantages of using natural polymers are their biocompatibility, nontoxicity and biodegradability [1].

Pectin is an anionic polysaccharide present in the cells of most plants [3]. It is mainly composed of linear chains containing α-(1→4)-D-galacturonic acid with carboxyl groups. The acid molecules are partially substituted with methyl ester or acetyl ester. For this reason, pectins are divided into the two groups of low or high methyl-esterified pectin, depending on the percentage of methylation of the carboxylic groups. Pectin containing a high amount of free carboxylic groups is defined as poly-anionic, low methyl-esterified pectin. The biopolymer can form a gel via cross-linking with calcium ions. The negatively charged carboxyl groups interact with the calcium ions. However, the type of pectin, its concentration and the cross-linking conditions influence the gel formation [4]. Hydrophobic interactions between the pectin chains and hydrogen bonding interactions between the amide groups contribute to the proposal of employing amidated low-methoxy pectins as very good natural polymers for bead formation [5].

The disadvantages of using pectin beads for drug delivery applications are related to the decomposition of the beads and the difficulty of controlling the release of the drug [6]. On the other hand, the synthetic polymers are intensively developed for pharmaceutical applications. The newly synthetized polymers may constitute a matrix for controlled delivery of variety of bioactive molecules, including cytostatics and other medicinal substances [7,8,9].

Compound 5-ASA (5-aminosalicylic acid, mesalazine) is the therapeutically active moiety in the treatment of inflammatory bowel disease. However, plain mesalazine is completely absorbed in the upper part of the intestine. Therefore, pharmaceutical formulations have been designed that can carry mesalazine undisturbed through the stomach, the duodenum and the proximal jejunum to deliver high concentrations of 5-ASA to the targeted inflammatory sites of the distal small intestine and colon [10,11].

Although several investigations on pectin beads containing the drug have been reported [12,13,14,15,16,17,18], a study on pectin formulations containing synthetic polymers is not found in the literature.

The aim of this study is to propose a formulation of pectin beads containing a synthetic polymer for colon-targeted drug delivery and to evaluate the release kinetics of the drug from the carriers. The incorporation of the synthetic polymer into the pectin formulation may overcome the difficulties associated with the fast decomposition of pectin beads and may prolong the release of the drug.

## 2. Results and Discussion

### 2.1. Morphological Studies

**Figure 1:**
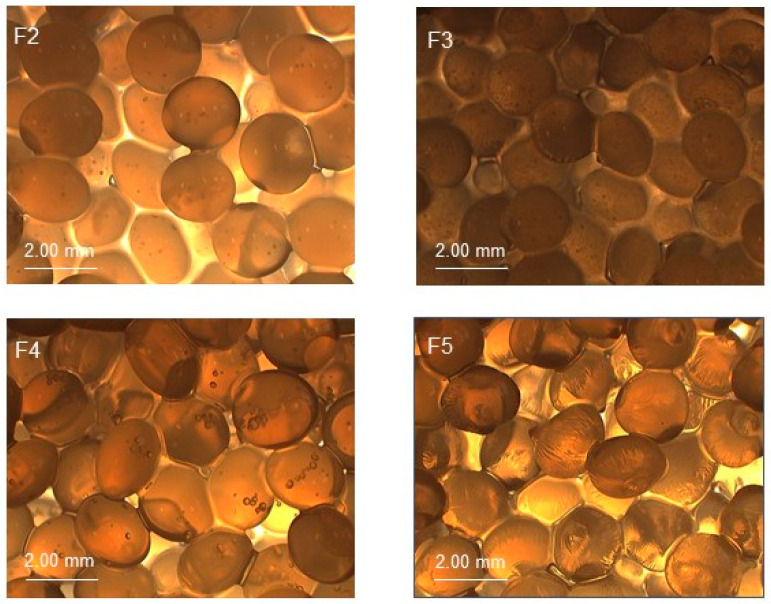
The microscopic pictures of millibeads F2–F5 unloaded with 5-ASA.

The picture of obtained millibeads doped with synthetic polymers before drying were illustrated in Figure 1. The images of all formulations are presented in supplementary materials in Figure S1. 

**Figure 2:**
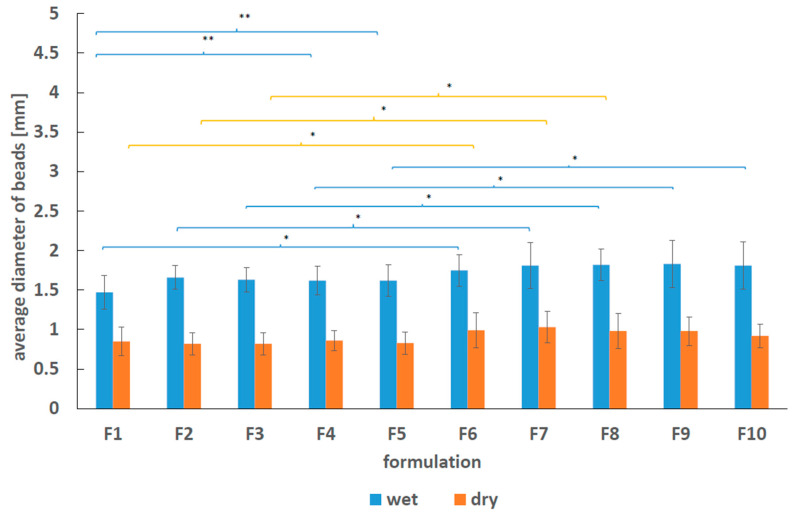
The average diameter of beads before and after drying. Significant differences between average diameter of beads, *p* < 0.05 (described by * for the influence of 5-ASA component, and by ** for the influence of the incorporation of synthetic polymer).

The obtained results of the mean diameter of the dry and wet formulations F1–F10 are shown in Figure 2.

Produced millibeads had spherical morphology, however the transparency was slightly different, what may implicate variability in the density, as well as light absorbance. The superficial morphology of the beads was smooth and they were resilient. The observed discrepancies of diameters of produced beads may be ascribed to the repulsive bonds between polymers. The interactions between chains may have the impact on the release of the drug due to steric hindrances of long polymeric chains. The diameters of the wet formulations ranged from 1.47 ± 0.21 mm to 1.83 ± 0.30 mm and were significantly higher than the diameters of the dry beads that were in the range from 0.82 ± 0.14 mm to 1.03 ± 0.20 mm. This difference was mainly related to the water loss that occurs during drying. 

The variability of bipolymeric beads diameters was analysed employing ANOVA combined with Tukey’s honest significant difference test (Tukey’s HSD test) and the statistical results are listed in Tables S1 and S2 attached in supplementary materials. The values of mean diameter of wet and dry beads was also presented in supplementary materials in Table S3. It was found that the mean values of the diameters of wet formulations F1–F5 were different from the mean values of the diameters of wet formulations F6–F10 loaded with 5-ASA. In the case of dry beads, the mean values of the diameters of formulations F1–F3 were slightly lower than the values obtained for formulations F6–F8. The differences can be explained by the incorporation of 5-ASA into formulations F6–F10. The dry F9–F10 beads did not changed the diameter comparing to the diameter of the beads unloaded with 5-ASA. The comparison of the mean diameters of the beads F1, and the mean diameters of the formulations containing synthetic polymers F2–F5 gave no substantial difference between the wet and dry preparations, although ANOVA test indicated the difference between wet F1 and F4, F5 millibeads. Comparing the mean diameters of F6 beads and F7–F10 doped with synthetic polymers the differences were not observed in the case of wet as well as dry formulations F7–F10. The incorporation of AX and PEG increased only wet beads diameters unloaded with 5-ASA.

### 2.2. Drug Release Study

**Figure 3:**
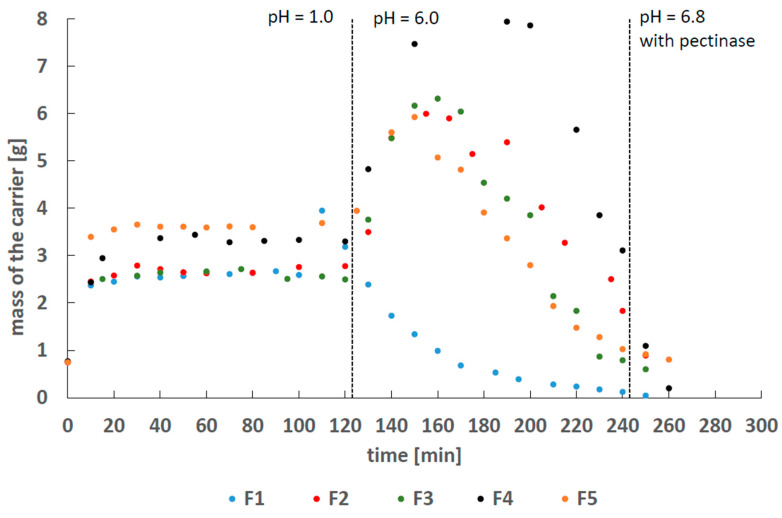
The mass variability of the carrier in time, in the solution of pH = 1, pH = 6.0 and pH = 7.4 with pectinase.

The drug was released into a pH = 7.4 buffer solution with pectinase that reflected the fluids in the colon [19]. The stability of pectin carriers F1–F5 in pH = 1, 6.0 and 7.4 with pectinase conditions simulating the gastric environment of gastrointestinal tract (GIT) was studied, and the results are shown in Figure 3. 

The acidic environment did not influence the decomposition of the pectin carrier. The observed initial increase in the matrix weight was caused by the penetration of water into the bead interior. These results suggest that pectin carriers can overcome the acidic stomach environment and reach the colon. The carriers decompose in the presence of an alkaline environment and intestinal bacteria. The disintegration of the carrier may then cause the release of the drug.

**Figure 4:**
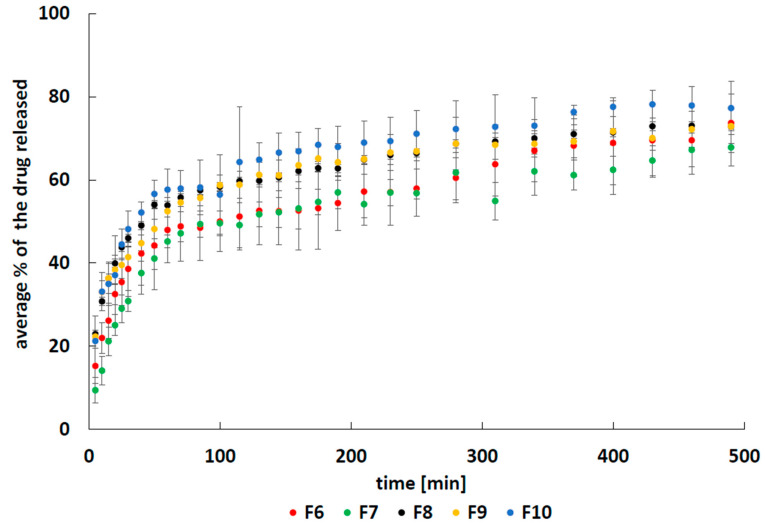
The release curves of 5-ASA from formulations F6–F10.

Several kinetic models, which are listed in Section 4.5, were used in the drug dissolution study. It was found that neither of the employed equations was appropriate to describe the dissolution profiles of 5-ASA in the 8-hour time period. The dissolution of the drug from the studied formulations was very fast at the beginning, followed by sustained drug release. The obtained release curves are shown in Figure 4.

The dried beads immersed in the acceptor fluid swelled during the initial stage, after which their volume became constant. This observation combined with the concentration gradient of the drug between the beads and the acceptor fluid could explain the characteristic release profile of 5-ASA from formulations F6–F10. The influence of the drug concentration gradient on the release was confirmed by the results obtained from the K-P equation. The derived values of the parameter n were below 0.5 in most cases, indicating that the mass transport occurred via diffusion. The value of n was 0.65 ± 0.08 only in the case of drug release from formulation F7, suggesting anomalous transport [20]. The results are consistent with results obtained by Shen et al. [21]. Their work revealed that the initial drug release was very fast, followed by a sustained drug release. This release pattern was explained by the electrostatic interaction between the drug and matrix as well as the concentration variability between the formulation interior and the bulk.

**Figure 5:**
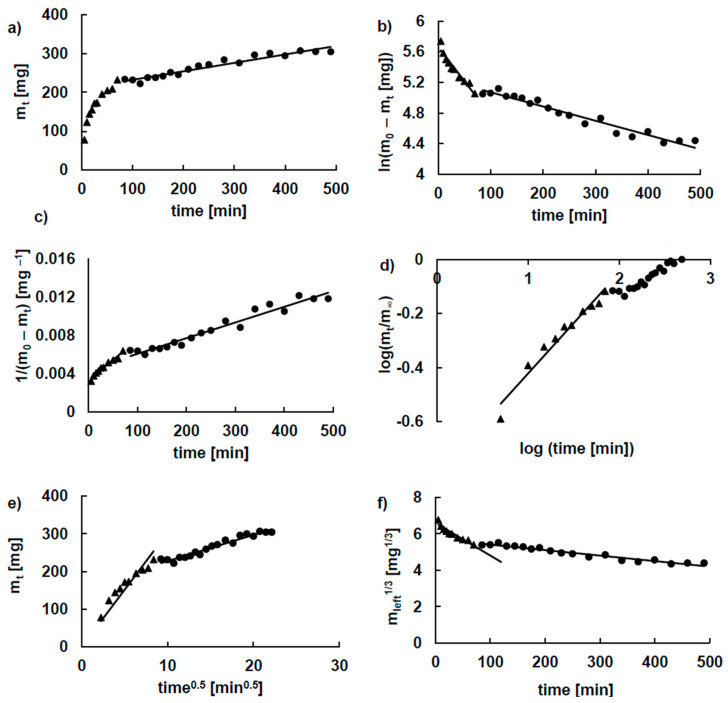
Kinetics of the release of 5-ASA from formulation F8 according to (**a**) zero-order, (**b**) first-order, (**c**) second-order, (**d**) Korsmeyer–Peppas (**e**) Higuchi (**f**) Hixon–Crowell model; experimental points of the first stage are marked ▲ and of the second stage ●, the line is the guide for the eye.

To carry out the kinetic analysis of the release of 5-ASA, the obtained dissolution profiles were divided into two stages. The first stage included the drug release as well as matrix swelling. The second stage contained only the dissolution of the drug. An example of fitting the experimental data of the release of 5-ASA from formulation F8 to the kinetic equations is shown in Figure 5. Based on this analysis, the kinetic parameters were derived, and they are listed in Table 1 and Table 2. Considering the values of the correlation coefficient R^2^, it was found that the first stage of the release of 5-ASA from formulations F6–F10 was mainly described by the Korsmeyer–Peppas or by the second-order kinetic models. The highest release rate constants calculated for this stage using the F-O, S-O, H and H-C equations were obtained for the release of 5-ASA from formulation F10. The shortest half release time was obtained for the 5-ASA release from formulation F10. The lowest t_0.5_ value was obtained from the K-P model for the drug release from formulation F8, and the highest value of the release rate constant was obtained for 5-ASA release from formulation F9. Summarizing these results, it can be concluded that the incorporation of the synthetic polymer into the formulation did not significantly prolong the release of 5-ASA from pectin beads during the first stage of the release. This may be explained by the dissolution of the drug from the surface immediately after immersion in the acceptor fluid.

The second stage of the release of 5-ASA from the studied formulations cannot be analyzed by the K-P model. This equation may be used only when m_t_/m_∞_ < 0.6 [20,22]. According to the Z-O, F-O, H, H-C kinetic models, the fastest 5-ASA release during second stage was observed for formulation F6, which did not contain the synthetic polymer. The fastest 5-ASA dissolution was observed for formulation F10 when the S-O equation was used, although the difference between the k_2_ values obtained for the drug release from formulations F10 and F6 was not large. The k_2_ values were 1.4 ± 0.4 mg^−1^ min^−1^ and 1.2 ± 0.2 mg^−1^ min^−1^ for the drug release from F10 and F6, respectively. The half release time was also the shortest for the dissolution of the drug from formulation F6. However, when the S-O equation was used, the shortest half release time was observed for the 5-ASA release from formulation F10. There was no substantial difference between the t_0.5_ obtained for the drug release from formulations F6 and F10. The t_0.5_ values for the drug release from F6 and F10 were 413.0 ± 69.0 min and 381.8 ± 130.5 min, respectively. These results suggest that incorporation of the synthetic polymers into the pectin beads prolonged the release of the drug. The mass transfer in formulation F6 that did not contain the second polymer was more efficient than the mass transfer in formulations F7–F10 with a high density of polymer chains hindering drug migration. The daily therapeutic dose of 5-ASA in the range of 1.5–4.5 g was applied in the active ulcerative colitis [23], and doses may be higher according to developed dosing schemes [24]. In the presented study, the level of 5-ASA used in one release test was 500 mg. The proposed dosing system is thus suggested to be used 3 times a day. Prospectively, the dosing may be increased to double the administered value of 5-ASA to 1000 mg. This form of the drug appears to be acceptable for patients with swallowing difficulties. The multi-compartmental form of the preparation may enable protection against an undesired increase in the drug concentration in the case of partial damage of coatings, as the dose was divided into tiny doses. The higher doses will be further developed. The release at 1.0 pH should be performed in further studies, to exclude enhanced loss of 5-ASA in gastric fluid in the preliminary phase of release. The resulting formulations after respective evaluation will be proposed for further in vivo experiments to evaluate the prospective clinical efficacy. 

### 2.3. The Difference Factor f_1_, the Similarity Factor f_2_

The influence of the incorporation of a synthetic polymer into the beads on the release behaviour of 5-ASA was evaluated using the difference factor f_1_ and the similarity factor f_2_. The calculated values of f_1_ and f_2_ are listed in Table 3. 

According to FDA recommendations [25], f_1_ values ranging from 0–15 and f_2_ values greater than 50 ensure the similarity or equivalence of two release curves. It was found that the incorporation of PVP or PEG 4000 into the pectin beads changed the release behavior of 5-ASA from the beads. The f_1_ values were higher than 15, and the f_2_ values were below 50, indicating the difference. The results were consistent with the kinetic analysis, which also revealed that the incorporation of a synthetic polymer in the pectin beads may prolong the release of 5-ASA. However, the incorporation of PA and AX into the pectin beads did not significantly influence the release behavior of the drug. The obtained values of f_1_ were below 15, and the values of f_2_ were above 50, suggesting that the dissolution process was similar.

A comparison of the release of 5-ASA from F7 containing PA with the release from F8, F9 and F10 revealed differences in the release behavior. The obtained f_1_ values were not in the range 0–15, with values of 27.27, 19.64, and 27.70, respectively. In the comparison of the drug release from F7 and F8 as well as that from F7 and F10, the variability in f_2_ was confirmed. In the comparison of the calculated f_2_ for the release of 5-ASA from F7 and F9, no difference was observed, although f_2_ was slightly above 50, with a value of 50.11. However, it is known that the similarity is higher for values closer to 100 [20]. Thus, it can be concluded that the release behavior of 5-ASA from the studied formulations was also dependent on the type of the synthetic polymer incorporated in the formulation.

### 2.4. Statistical Analysis

The influence of the variability of formulations on the release of 5-ASA was studied by employing one-way analysis of variance (ANOVA). This test combined with Tukey’s honest significant difference test (Tukey’s HSD test) can be used when the impact of one factor on the results of the conducted research is analyzed [26]. The data obtained in the present work are summarized in Table 4. 

The only difference was noticed in the comparison of the dissolution profiles of the drug from formulations F7 and F10. Both formulations contained the synthetic polymer PA or PEG 4000, indicating that the synthetic polymer type influenced the drug release. The same difference was observed from the analysis of the f_1_ and f_2_ factors. However, in the comparison of the different formulations, both factors indicated a difference in the drug release that was not found using ANOVA. It is worth noting that ANOVA was not mentioned in any of the FDA documents, although this method was considered useful in previous studies [20,22,26],. The use of ANOVA improves knowledge of the dissolution process of controlled-release formulations.

### 2.5. FTIR Spectroscopy

**Figure 6:**
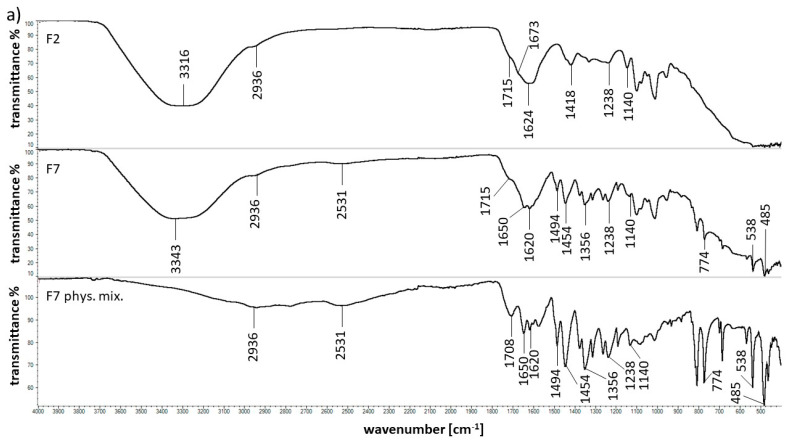

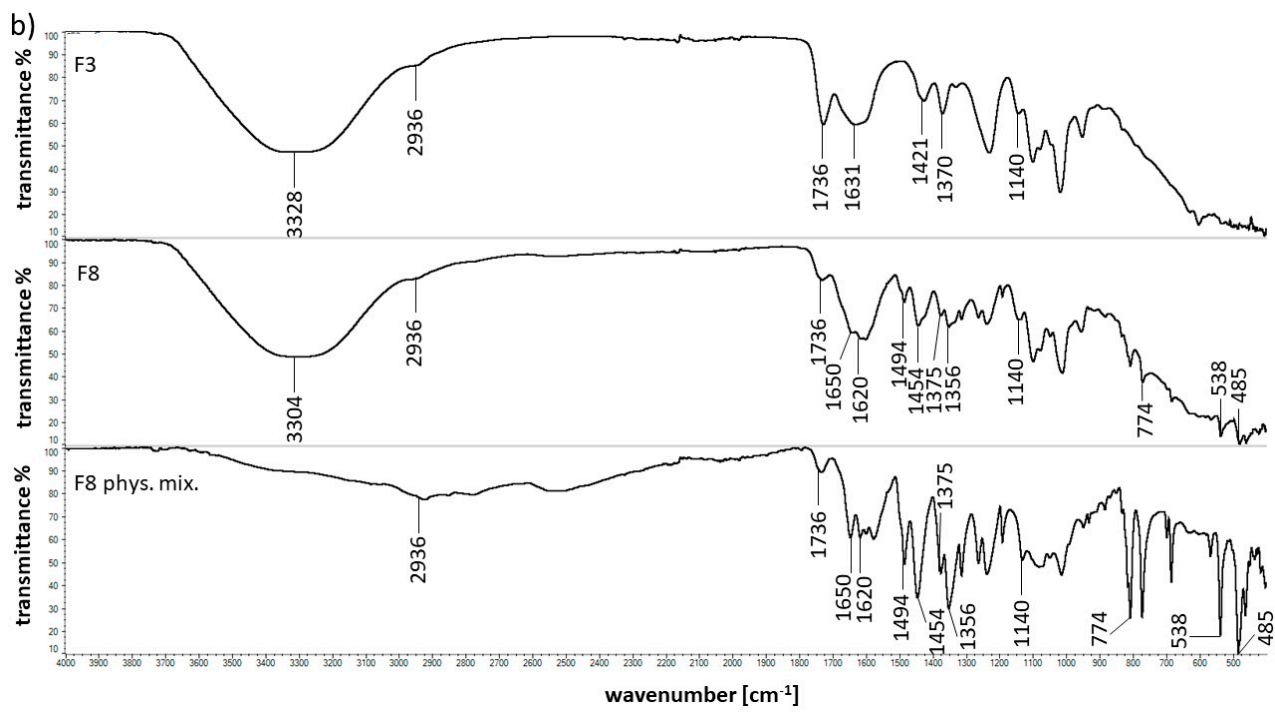

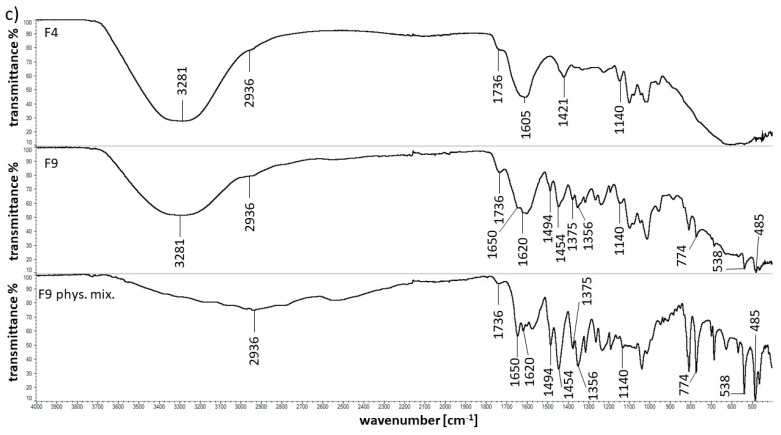

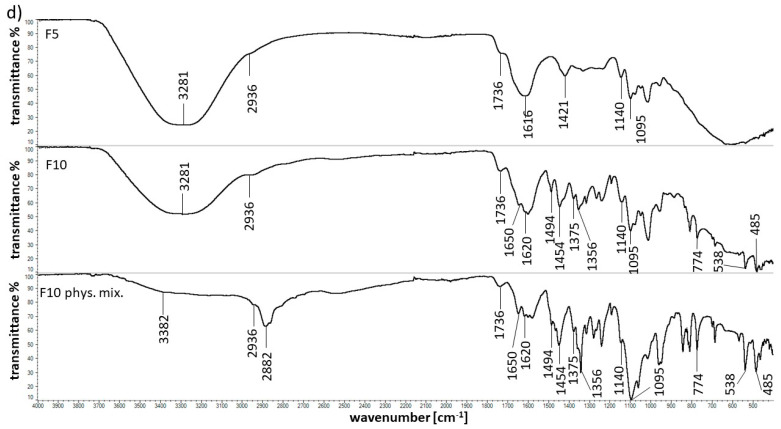
The FTIR spectra of the formulation of (**a**) F2, F7 and its physical mixture; (**b**) F3, F8 and its physical mixture (**c**) F4, F9 and its physical mixture; (**d**) F5, F10 and its physical mixture.

To evaluate the interactions between the drug and the natural polymer as well as the interactions between the drug and the synthetic polymer, the FTIR spectra of the pure components and formulations F1–F10 as well as their physical mixtures were recorded. The most interesting spectra are shown in Figure 6. The measured FTIR spectra of 5-ASA showed a broad absorption band at 2773 cm^−1^ attributed to the stretching vibration of the OH group. According to Mladenovska et al. [27] and Hu et al. [28], the characteristic bands at 1650 cm^−1^ belonging to the C=O group, 1620 cm^−1^ assigned to the N-H group and 1356 cm^−1^ corresponding to C-N stretching were observed. The characteristic bands of 5-ASA at 1494, 1454, 774, 538, and 485 cm^−1^ were also observed. All maxima were found in the spectra of formulations F6–F10 as well as in the spectra of their corresponding physical mixtures. These results suggest the absence of an interaction between the drug and the polymers. Neufeld et al. [13] also reported that there was no interaction between 5-ASA and pectin in their physical mixture.

The characteristic APN bands were described in our previous work and were located at 2936, 1736, 1673, 1596, 1421, and 1140 cm^−1^ [29]. The bands at 2936, 1736, and 1140 cm^−1^ were found in the spectra of formulations F1–F10 and their physical mixtures, although in the spectra of F2, F7 and its physical mixture, the maximum at 1736 cm^−1^ associated with the C=O stretching vibration of the ester was shifted to 1715 cm^−1^. Moreover, in the spectrum of formulation F2 in Figure 6a, a band at 1624 cm^−1^ appeared, and simultaneously, the intensive band at 1697 cm^−1^ assigned to the PA carboxyl group was not present. This observation was similar to results in our previous work [29] and the study of Alvarez-Gayosso et al. [30]. The vibrations at approximately 1700 cm^−1^ belonging to the -COOH group could decrease in intensity upon the appearance of the maximum in the range of 1650 to 1500 cm^−1^ attributed to COO^−^ stretching. This observation suggests that there was an interaction between the APN carboxylate group and PA carboxyl group. The possibility of this interaction was confirmed by the disappearance of the PA band at 1451 cm^−1^ and a shift in the PA maximum at 1227 cm^−1^ to 1238 cm^−1^ in the spectra of F2, F7 and its physical mixture. The interaction between pectin and another polymer, brea gum, was studied by Slavutsky et al. [31]. It was observed that the band of the -OH group and asymmetric COO^−^ stretching vibrations were shifted to lower frequencies, indicating the formation of hydrogen bonds between both polymers. A similar interaction was also found between pectin and sodium alginate [32]. The observed interaction between APN and PA may be responsible for the discrepancy in the dissolution study from F7 and F10 obtained from ANOVA test. It was mentioned that the comparison of the release of 5-ASA from formulations doped with synthetic polymers indicated the differences between F7 and F10. However, comparing the release from F6 and F7 based on f_1_ and f_2_ values, no variability was noticed.

Carboxyl-carboxylate proton binding was also observed in previous studies [33,34,35,36]. The existence of hydrogen bonds between two carboxyl groups O―H····O was found. The band of PA at 1412 cm^−1^ was shifted to 1418 cm^−1^ in the spectrum of F2. However, in the spectra of F7 and its physical mixture, the peak at 1412 cm^−1^ was not visible. This may indicate an interaction between PA and 5-ASA because 5-ASA was absent in formulation F2. In addition, the interaction between the carboxyl group of PA and the carboxyl group of the drug-salicylic acid was proposed in our previous work [29]. Since 5-ASA is the amine derivative of salicylic acid, a similar effect is also possible in the case of 5-ASA and PA.

The APN peak at 1673 cm^−1^ was very weak in the spectra of formulations F1 and F2 and was not found in the spectra of F3–F10 and their corresponding physical mixtures. The analysis of the spectra of F1–F5 revealed that the APN maximum at 1596 cm^−1^ was shifted to 1600, 1624, 1631, 1605 and 1616 cm^−1^ and was not present in the spectra of formulations F6–F10 and their physical mixtures. These observations may be due to an interaction between APN carboxyl groups and calcium ions. The peaks in the FTIR spectrum of calcium chloride observed at 1628 and 1613 cm^−1^ disappeared in the spectra of formulations F1–F10 and their physical mixtures, confirming the interaction of the APN carboxyl group with calcium ions.

The FTIR spectrum of PVP was discussed in previous investigations [37,38]. It is interesting to note that the characteristic band at 1661 cm^−1^ assigned to the C=O stretching vibration was not present in the spectra of F3, F8 and the physical mixture of F8 (Figure 6b). It should be mentioned that in the spectrum of F3, the wide band at 1631 cm^−1^ was observed and was difficult to explicitly assign to any of the F3 components. According to the investigation of Alvarez-Gayosso et al. [30], vibration bands assigned to carboxylic acid, -COOH, appear at approximately 1700 cm^−1^, although the carboxylate ion -COO^−^ vibrations are located in the range from 1650 to 1500 cm^−1^. This observation indicates an interaction between the PVP carbonyl group and APN carboxylate group. Additionally, the PVP neighboring peaks at 1496 and 1463 cm^−1^ were not observed in the spectra of F3, F8 and the physical mixture of F8. These results may confirm the interaction between both polymers. Wu et al. [39] found an interaction between the hydroxyl group on the carboxyl of PA (-COOH) and the oxygen lone pair of the carbonyl group of PVP (C=O).

Moreover, it was noticed that the PVP bands located at 1425 and 1370 cm^−1^ were not found in the spectra of F8 and its physical mixture, suggesting an interaction between PVP and 5-ASA because the maxima were present in the spectrum of formulation F3, into which no 5-ASA was incorporated. This FTIR data were consistent with the results obtained from release study. The parameter f_1_ and f_2_ indicated the difference in the dissolution of 5-ASA from F6 and F8. In the F6 formulation, no interaction between APN and 5-ASA was observed, but in F8 the interaction between the doped polymer and the drug was noticed.

The FTIR spectra of formulations F4 and F9 containing AX were illustrated in Figure 6c. The analysis revealed that the characteristic maxima of AX at 1640, 1544, 1440, 1388, and 1176 cm^−1^ were not found in the spectra of F4, F9 and its physical mixture. The -C=O group exists in the structure of AX, as in the case of PA and PVP. The carbonyl groups of AX may bind to the carboxyl groups of APN or 5-ASA. This observation is consistent with the results obtained from the FTIR results of F3 and F8 formulations containing PVP.

The obtained FTIR spectrum of PEG 4000 has characteristic bands at the frequencies of 3447, 2882, and 1095 cm^−1^, which is in agreement with previous investigations [40,41]. All the maxima were also found in the spectrum of the physical mixture of F10 shown in Figure 6d. However, the weak band at 3447 cm^−1^ was shifted to 3382 cm^−1^ in the spectrum of the physical mixture of F10. In the case of the spectra of formulations F5 and F10 (Figure 6d), the maxima at 3447 and 2882 cm^−1^ were not found. This may be explained by an interaction of the terminal -OH group belonging to PEG 4000 with hydroxyl group belonging to APN. This observation may also be due to the presence of water molecules in formulations F5 and F10, which has a broad band assigned to the OH group. The hydrogen from one molecule may bind the oxygen from the other moiety and form the hydrogen bond. The signal at 1095 cm^−1^ observed in the spectra of formulations F5, F10 and the physical mixture of F10 may be assigned to the interior -C-O-C- ether group of PEG [42]. The hydrogen bond may explain the difference in the release pattern of 5-ASA from F10 in comparison to the release from F6. 

It is worth mentioning that the FTIR spectra of all studied formulations showed a broad band in the range of 3000–3600 cm^−1^ related to the existence of free or bound OH groups.

To conclude, the FTIR study revealed possible interactions between pectin and the synthetic polymers as well as the polymer and the drug. These results may explain the variability in the drug release from formulations F6–F10 containing different synthetic polymers.

### 2.6. DSC Study

**Figure 7:**
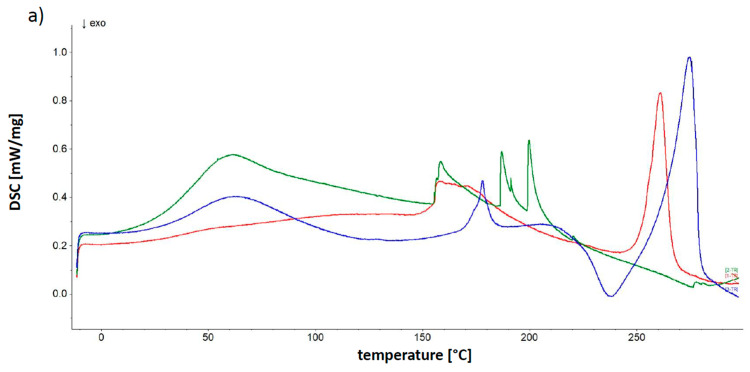

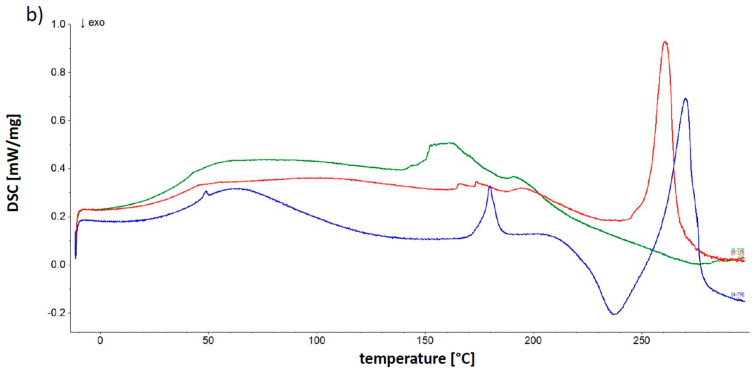

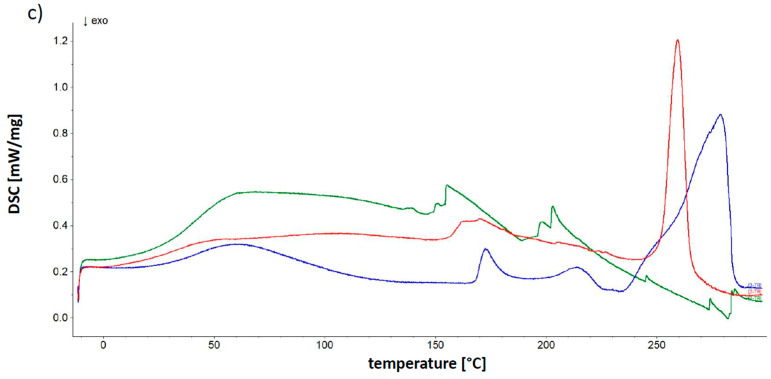

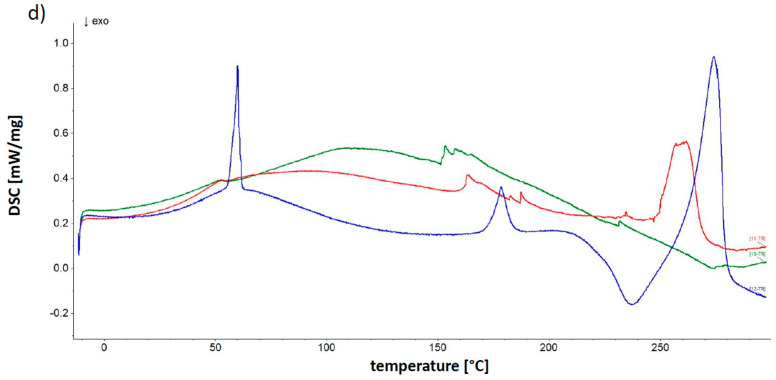
The DSC thermograms of (**a**) F2 (green line), F7 (red line) and physical mixture of F7 (blue line); (**b**) F3 (green line), F8 (red line) and physical mixture of F8 (blue line); (**c**) F4 (green line), F9 (red line) and physical mixture of F9 (blue line); (**d**) F5 (green line), F10 (red line) and physical mixture of F10 (blue line).

The thermograms of formulations containing synthetic polymer, as well as their physical mixtures are shown in Figure 7, and the temperatures of obtained maxima are collected in Table 5. The results allowed us to study the interaction between the formulations components. The measured DSC curve of pure 5-ASA revealed a sharp endothermic peak at 284.1 °C related to its melting point. This value was slightly higher comparing to the literature values of 270.0 and 277.5 °C obtained by Neufeld et al. [13] and Hu et al. [28], respectively. The peak of 5-ASA was found in the DSC thermograms of formulations F7–F10 containing the drug as well as on their physical mixtures and were presented in Figure 7. The maximum on the curves of physical mixtures was in the range 270.1–278.4 °C and corresponded to the literature data [13,28], and was slightly shifted to lower temperatures 259.2–263.5 °C in the case of formulations F7–F10. These results indicate no interaction between the drug and polymers.

The two endothermic events of CaCl_2_ obtained at 116.9 and 138.7 °C were not found in the thermograms of formulations F1–F10. Furthermore, the two maxima of CaCl_2_ did not exist in the thermograms of their physical mixtures.

The obtained APN thermogram was similar to the thermograms shown in our previous study [29]. The thermal profile of APN revealed an endotherm at 73.0 °C assigned to the evaporation of water and two endothermic peaks at 178.9 and 192.8 °C, as well as an exothermic process at 236.2 °C. 

The APN endotherm at 178.9 °C was found in the plots of formulations F7–F9 and physical mixtures M_F7_–M_F10_ in the range 169.5–179.9 °C, although it was not observed in the thermograms of formulations F2–F5 not containing the drug. The weak signal at 192.8 °C was present in the curves of physical mixtures M_F7_–M_F10_ between 206.2 and 213.6 °C and formulations F3, F4, F8 from 192.0 to 197.8 °C. The exotherm at 234.5 °C assigned to APN was found in the thermal profiles of physical mixtures M_F7_–M_F10_ and were located at 224.0–238.4 °C. However, this signal was not found in thermograms of formulations F2–F10.

From the measured thermal profile of PA two endothermic processes were located at 62.4 and 221.5 °C. In Figure 7a both maxima of PA at 62.2 and 206.2 °C were noticed in the thermogram of the physical mixture of F7. However, the peak at 206.2 °C may be also assigned to the shifted signal of APN at 192.8 °C. In the thermogram of F2, the endoderm was noticed at 61.1 °C. Neither the maximum at 62.4 °C nor the signal at 221.5 °C was observed in the thermogram of F7.

The thermograms of F3, F8 doped with PVP, and its physical mixture were presented in Figure 7b. The thermal behavior of PVP revealed an endothermic peak at 47.1 °C coincided with the reported endothermic process of melting [43]. The signal assigned to PVP was present in all thermograms in Figure 7b in the range 46.1–49.3 °C. 

In Figure 7c, the thermal profiles of formulations F4, F9 doped with AX as well as M_F9_ were presented. The obtained AX thermogram showed several broad endothermic events at 57.7, 144.0, 211.3, and 248.8 °C and exothermic events at approximately 122.1, 182.3, and 266.9 °C. The maxima belonging to AX were hardly noticeable in the curves in Figure 7c. These results may suggest the interaction between AX and the components of formulations.

The obtained PEG 4000 thermal profile was similar to the curve from the literature and showed a sharp endothermic maximum at 58.4 °C, and a broad exothermic process at approximately 206.7 °C [40,44]. The thermograms of F5, F10 containing PEG 4000 and physical mixture of F10 were shown in Figure 7d. The sharp endotherm at about 52.0–60.0 °C coming from PEG 4000 was observed in all profiles. The exotherm of PEG400 at 206.7 °C was not observed in Figure 7d.

Apart from maxima that were assigned to the components of formulations, broad sharp endothermic processes appeared in the range of approximately 152.8–169.5 °C. The new signals were observed in the curves of formulations, although they did not exist in the profiles of physical mixtures. The maxima, together with the absence of signals of CaCl_2_ as well as the lack of the exotherm of APN may indicate the interaction between calcium ions and APN. Moreover, the disappearance or shifting of APN endotherms, the absence of PA maximum at 221.5 °C in formulation F2 and F7 and a new peaks at 186.3 and 199.1 °C in the thermogram of formulation F2 may be a result of the interaction between the natural and the synthetic polymer. The lack of signals of AX in Figure 7c and the appearance of the new endotherm at 202.2 °C in the curve of F4, as well as the absence of the exotherm of PEG400 at 206.7 °C in Figure 7d may be also explained by the bond formation between polymers. The DSC study is consistent with the data from the spectroscopic analysis suggesting the interaction between components.

According to Buning et al. [45] numerous preparation proposed in the Crohn’s disease have been unrecommended by the medical authorities due to the lack of efficacy or severe side effects. However, 5-ASA is used extensively in considered condition. The presented millibeads may enable achievement of the appropriate 5-ASA concentration in the distal sections of the GIT. The bipolymeric beads are supposed to be protective to the drug, in terms of unfavourable absorption in the stomach.

## 3. Materials

Amidated pectin (APN) was obtained from C&GSp. z o.o. (Jasło, Poland). CaCl_2_, NaOH and KH_2_PO_4_ were derived from Chempur (Piekary Śląskie, Poland). Polyvinylpyrrolidone (PVP) was obtained from BASF (Ludwigshafen, Germany), polyacrylic acid (PA) was obtained from LUBRIZOL (Wickliffe, OH, USA), and ammonium acryloyldimethyltaurate (AX) was obtained from CLARIANT (Muttenz, Switzerland). Polyethylene glycol (PEG) 4000 was a gift from the local company Hasco-Lek (Wroclaw, Poland). 5-Aminosalicylic acid (5-ASA) was supplied from Sigma-Aldrich (St. Louis, MO, USA). Pectinase was obtained from a local market (activity of 4160 PE/mL, BIOWIN, Łódź, Poland). All synthetic chemicals were of analytical reagent grade and used without further purification.

## 4. Methods

### 4.1. Preparation of Pectin Beads

Pectin beads were prepared using the ionotropic gelation technique [12,14,46]. The composition of formulations F1–F10 was presented in Table 6. The ingredients were dissolved in distilled water. The concentration of APN was 4.5% (*w/v*) in formulations F1 and F6. In the other formulations, the APN concentration was 3.6% (*w/v*), and the concentration of the synthetic polymer was 1.0% (*w/v*). The concentration of 5-ASA in formulations F6–F10 was 2.3% (*w/v*). A homogenizer was employed to obtain a homogeneous mixture. The mixture was dropped to 500 mL of a gently agitated 2% calcium chloride solution (magnetic stirrer MIXdrive 6: 2 mag magnetic ^e^motion, Muenchen, Germany) via a 20 G needle connected by a tube. A flow rate of 20 mL/min was used by employing a peristaltic pump (PS-16 Sipper Pump, PG Instruments Limited, Leicestershire, UK). The obtained beads were separated by filtration using a sieve, washed with distilled water and dried at 50 °C.

### 4.2. Morphological Studies

The diameters of beads F1–F10 before and after drying were obtained using a microscope (Motic SMZ-171, Xiamen, China) equipped with a digital camera (Moticam, Xiamen, China) connected to the computer with the software Motic Images Plus 2.0. The diameter of 100 beads was measured from the images by the software, and the mean values of the dry and wet diameters were calculated for each formulation.

### 4.3. FTIR Spectroscopy

The FTIR spectra were recorded using a Thermo Scientific Nicolet iS50 FT-IR Spectrometer (Waltham, MA, USA) in the wavenumber range of 500–4000 cm^−1^. The pure components such as APN, 5-ASA, PA, PVP, AX and PEG 4000 as well as formulations F1–F10 and their corresponding physical mixtures were measured. The samples of formulations F1–F10 were measured in the wet state because the spectra of the dry beads F6–F10 were noisy.

### 4.4. DSC Study

The DSC study was carried out employing a differential scanning calorimeter (DSC 214 Polyma, Netzsch, Selb, Germany). The thermograms of the dry beads and 5-ASA as well as formulations F1–F10 and their corresponding physical mixtures were obtained. The DSC aluminum crucibles with an aluminum lid were filled with 3 mg of sample. The measurements were conducted with a nitrogen flow rate of 50 mL/min at a heating rate of 5 °C/min in the temperature range of −10–300 °C.

### 4.5. Release Studies

The stability of pectin carriers F1–F5 was evaluated in pH = 1.0, 6.0, and 7.4 with pectinase. The samples of millibeads were weighted and placed on the sieve which was introduced into a buffer of pH = 1.0 for 2 h; the mass decrease was recorded every 15 min—the samples were removed from the solution in defined time intervals, dried using paper towels and weighted. Afterwards, the millibeads were transferred to the buffer of pH = 6.0 for next 2 h and the same procedure was employed. Finally, samples were placed in the buffer pH = 7.4 with pectinase.

The release of 5-ASA from formulations F6–F10 was carried out using the dissolution basket apparatus 1 (Erweka DT126/128 light, Heusenstamm, Germany) with a rotation speed of 50 rpm at a temperature of 37 ± 0.5 °C [47]. The drug was released into 1 L of a pH = 7.4 buffer solution with 5 mL of added enzyme-pectinase to reflect the colon environment. The 3 mL samples were collected at appropriate time intervals within an 8-hour time period. The samples were replenished with pH = 7.4 buffer. All formulations were tested six times.

**Figure 8:**
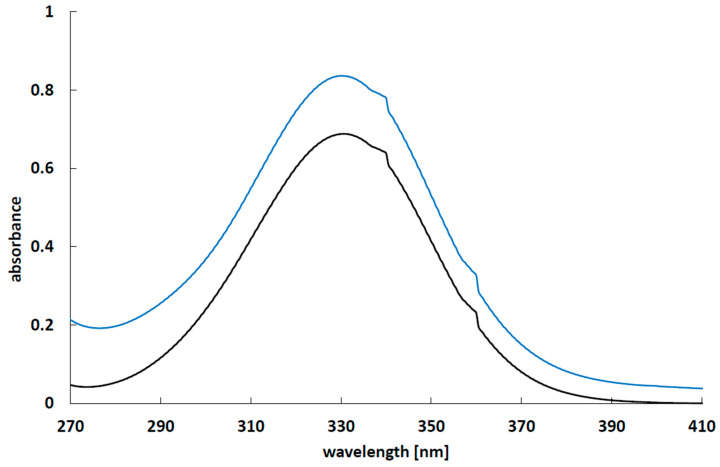
The UV-Vis spectrum of 5-ASA obtained at room temperature in buffer solution pH = 7.4, the concentration of 5-ASA was 0.03 mg/mL (black line) and in 2% calcium chloride solution, the concentration of 5-ASA was 0.05 mg/mL (blue line).

The UV-Vis spectra of 5-ASA in the pH = 7.4 buffer solution as well as in 2% calcium chloride solution were recorded employing a UV-Vis spectrophotometer (Jasco V-530, Tokyo, Japan) and are shown in Figure 8. 

The absorbance of all samples was read at a wavelength of 330 nm. At this wavelength, the absorbance maximum of 5-ASA in the buffer as well as that in the 2% calcium chloride solution were observed. The amount of the released 5-ASA (or the amount of the drug washed out to the calcium chloride solution from the beads) was calculated based on the prepared calibration curves. The obtained results were analyzed by zero-, first-, and second order kinetics and Higuchi [48], Korsmeyer–Peppas [48,49], and Hixon Crowell [50] models according to the equations shown in Table 7. The release parameters, including the release rate constants, the half-release time, and the diffusion coefficient n, were calculated. The correlation between the experimental data and the theoretical curve was described by the correlation coefficient value (R^2^). Based on the value of R^2,^ the appropriate kinetic models were selected.

### 4.6. The Difference Factor f_1_, the Similarity Factor f_2_

The difference factor f_1_ and the similarity factor f_2_ were calculated as recommended by the FDA [25,26]:

(1) $$f_{1}=\frac{\sum_{t=1}^{n}\left|R_{t}-T_{t}\right|}{\sum_{t=1}^{n}R_{t}}\times 100$$

(2) $$f_{2}=50\times \log\left\{{\left[1+\frac{\sum_{t=1}^{n}{\left(R_{t}-T_{t}\right)}^{2}}{n}\right]}^{-0.5}\times 100\right\}$$

where n is the number of time points, R_t_ is the released value of the reference batch at time t, T_t_ is the released value of the test batch at time t.

### 4.7. Statistical Analysis

One-way analysis of variance (ANOVA) with Tukey’s test were employed to determine the significant differences between mean diameters of beads (n = 45).

The linearity of the kinetic models was carried out based on the least-squares regression method. The comparison of the correlation coefficient, R^2^, indicated the kinetic model that describes the observed processes well.

Statistical analysis of the release profiles was also conducted by ANOVA with Tukey’s test. It was established that a variability between two dissolution profiles occurred for values of probability p lower than 0.5 [20].

## 5. Conclusions

In summary, the present study revealed that the incorporation of a synthetic polymer into pectin beads prolonged the release of the drug, particularly during the second stage of the release process. A spectroscopic analysis combined with a DSC study found that there may be an interaction between APN and the synthetic polymers that may affect the dissolution of the active substance. The inclusion of a synthetic polymer into pectin beads improved the strength of the calcium ion cross-linked network. Additionally, in some cases, the FTIR results suggested an interaction between 5-ASA and the polymer that may also influence the dissolution of the drug. The calculated values of the factors f_1_ and f_2_ confirmed a difference in the release of 5-ASA when a synthetic polymer was incorporated into the formulation.

## Figures and Tables

**Table 1:** The obtained kinetic parameters of the first stage of the release.

Kinetic Model	Kinetic Parameters	F6	F7	F8	F9	F10
F-O	k_1_ × 10^3^ [min^−1^]	7.8 ± 1.9	8.3 ± 1.3	8.2 ± 0.2	7.2 ± 1.5	9.6 ± 2.7
	R^2^	0.87 ± 0.08	0.95 ± 0.05	0.88 ± 0.04	0.9 ± 0.1	0.85 ± 0.1
	t_0.5_ [min]	101 ± 29	84 ± 13	88 ± 23	98.9 ± 24.6	73.3 ± 21.5
S-O	k_2_ × 10^5^ [mg^−1^ min^−1^]	3.3 ± 0.6	3.3 ± 0.4	3.9 ± 1.0	3.4 ± 0.6	4.6 ± 1.1
	R^2^	0.93 ± 0.09	0.97 ± 0.04	0.90 ± 0.06	0.9 ± 0.1	0.86 ± 0.12
	t_0.5_ [min]	89 ± 18	84 ± 10	63 ± 14	83.6 ± 21.3	60.8 ± 17.1
H	k_H_ [mg × min^−1/2^]	24.9 ± 1.9	21.3 ± 1.2	30.3 ± 2.6	27.5 ± 2.6	31.0 ± 2.7
	R^2^	0.7 ± 0.2	0.93 ± 0.09	0.72 ± 0.20	0.66 ± 0.19	0.69 ± 0.19
	t_0.5_ [min]	64.7 ± 9.7	77.7 ± 8.4	42.8 ± 7.5	46.7 ± 8.7	39.4 ± 7.1
K-P	k_K-P_ × 10^2^ [min^−n^]	9.7 ± 2.4	4.8 ± 1.5	20.3 ± 4.3	20.8 ± 5.4	14.4 ± 7.2
	n	0.50 ± 0.07	0.65 ± 0.08	0.35 ± 0.06	0.30 ± 0.07	0.42 ± 0.12
	R^2^	0.96 ± 0.09	0.96 ± 0.08	0.94 ± 0.04	0.90 ± 0.07	0.85 ± 0.13
	t_0.5_ [min]	30 ± 17	37 ± 19	15 ± 7	18 ± 15	21.2 ± 25.5
H-C	k_H-C_ × 10^2^ [mg^1/3^min^−1^]	1.6 ± 0.4	1.8 ± 0.3	1.6 ± 0.5	1.4 ± 0.3	1.9 ± 0.6
	R^2^	0.86 ± 0.08	0.94 ± 0.05	0.87 ± 0.04	0.88 ± 0.12	0.84 ± 0.11
	t_0.5_ [min]	104 ± 31	85.0 ± 14.7	94.7 ± 26.3	105.7 ± 28.0	79.9 ± 24.6
Best fit		K-P	S-O	K-P	F-O, S-O, K-P	S-O

F-O—first order, S-O—second order, H—Higuchi model, K-P—Korsmeyer–Peppas model, H-C—Hixon–Crowell model.

**Table 2:** The obtained kinetic parameters of the second stage of the release.

Kinetic Model	Kinetic Parameters	F6	F7	F8	F9	F10
Z-O	k_0_ × 10 [mg min^−1^]	2.4 ± 0.4	1.7 ± 0.5	1.6 ± 0.4	1.4 ± 0.4	1.8 ± 0.6
	R^2^	0.89 ± 0.04	0.72 ± 0.20	0.74 ± 0.25	0.77 ± 0.13	0.64 ± 0.32
	t_0.5_ [min]	383.5 ± 60.3	541.6 ± 182.5	836.4 ± 355.7	816.6 ± 230.7	1061.4 ± 1091.6
F-O	k_1_ × 10^3^ [min^−1^]	1.7 ± 0.3	1.1 ± 0.3	1.2 ± 0.3	1.1 ± 0.3	1.6 ± 0.5
	R^2^	0.88 ± 0.05	0.71 ± 0.20	0.73 ± 0.26	0.76 ± 0.15	0.65 ± 0.35
	t_0.5_ [min]	451.5 ± 75.1	675.9 ± 238.0	705.7 ± 327.7	677.6 ± 184.1	724.3 ± 961.6
S-O	k_2_ × 10^5^ [mg^−1^ min^−1^]	1.2 ± 0.2	0.73 ± 0.27	1.0 ± 0.3	0.9 ± 0.3	1.4 ± 0.4
	R^2^	0.87 ± 0.06	0.59 ± 0.21	0.58 ± 0.3	0.67 ± 0.18	0.74 ± 0.36
	t_0.5_ [min]	413.0 ± 69.0	742.1 ± 358.9	1005.1 ± 641.4	716.5 ± 240.9	381.8 ± 130.5
H	k_H_ [mg × min^−1/2^]	7.5 ± 1.3	5.2 ± 1.6	5.1 ± 1.1	4.4 ± 1.0	5.8 ± 1.7
	R^2^	0.89 ± 0.04	0.71 ± 0.19	0.76 ± 0.24	0.81 ± 0.12	0.66 ± 0.31
	t_0.5_ [min]	89.5 ± 27.5	193.8 ± 146.4	651.0 ± 694.9	477.0 ± 247.0	1505.5 ± 3641.3
H-C	k_H-C_ × 10^3^ [mg^1/3^min^−1^]	2.9 ± 0.5	1.9 ± 0.6	2.1 ± 0.5	1.8 ± 0.5	2.5 ± 0.8
	R^2^	0.89 ± 0.04	0.71 ± 0.20	0.73 ± 0.26	0.77 ± 0.14	0.65 ± 0.34
	t_0.5_ [min]	440.1 ± 71.3	648.3 ± 223.9	673.7 ± 303.2	651.0 ± 175.4	674.6 ± 781.9
Best fit		Z-O, H, H-C	Z-O	H	H	S-O

Z-O—zero order, F-O—first order, S-O—second order, H—Higuchi model, H-C—Hixon–Crowell model.

**Table 3:** The values of the difference factor f_1_ and the similarity factor f_2_ obtained by comparing the release profile of 5-ASA from formulations F6–F10.

Factors of Difference and Similarity	Formulations	F7	F8	F9	F10
f_1_	F6	7.26	19.92	12.15	19.59
	F7	―	21.27	19.64	27.70
	F8	―	―	2.70	6.22
	F9	―	―	―	7.45
f_2_	F6	66.49	48.53	57.34	48.99
	F7	―	48.53	50.11	43.38
	F8	―	―	81.30	69.64
	F9	―	―	―	65.74

**Table 4:** The values of Tukey’s HSD test.

Formulations	F7	F8	F9	F10
F6	3.25	6.94	6.15	10.01
F7	―	10.18	9.40	13.26
F8	―	―	0.78	3.08
F9	―	―	―	3.86

**Table 5:** The obtained DSC maxima of formulations F2–F5 and F7–F10 doped with synthetic polymer, and respective physical mixtures (M_F7_–M_F10_), abbreviation described in the text.

F2	F7	M_F7_	F3	F8	M_F8_	F4	F9	M_F9_	F5	F10	M_F10_
―	261.2	274.7	―	260.6	270.1	―	259.2	278.4	―	263.5	274.2
―	171.3	178.1	―	173.7	179.9	―	169.5	172.7	―	―	178.5
―	―	206.2	192.0	194.4	206.4	197.8	―	213.6	―	―	206.8
―	―	238.4↓	―	―	238.1↓	―	―	224.0↓	―	―	237.5↓
61.1	―	62.2	49.3	46.1	48.7	―	―	―	52.0	52.0	60.0
158.3	159.4	―	154.4–161.5	166.1	―	154.0	159.8–169.5	―	152.8	162.3	―
186.3	―	―	―	―	―	―	―	―	―	187.4	―
199.1	―	―	―	―	―	202.2	―	―	―	―	―

**Table 6:** Composition of pectin beads.

Formulation	F1	F2	F3	F4	F5	F6	F7	F8	F9	F10
Natural polymer	APN	APN	APN	APN	APN	APN	APN	APN	APN	APN
Synthetic polymer	―	PA	PVP	AX	PEG 4000	―	PA	PVP	AX	PEG 4000
Active substance	―	―	―	―	―	5-ASA	5-ASA	5-ASA	5-ASA	5-ASA

APN—amidated pectin, PA-polyacrylic acid, PVP—kollidon, Polyvidone, Polyvinylpyrrolidone, Povidone, AX—Aristoflex, PEG—polyethylene glycol, 5-ASA-5—aminosalicylic acid, mesalazine.

**Table 7:** Kinetic equations used in the drug release analysis.

Kinetic Model	Equation
Zero-order	$m_{t}{=m}_{b}{+k}_{0}t$
First-order	$\ln\left(m_{0}{-m}_{t}\right)=\ln\left(m_{0}\right){-k}_{1}t$
Second-order	$\frac{1}{\left(m_{0}{-m}_{t}\right)}=\frac{1}{m_{0}}{-k}_{2}t$
Higuchi	$m_{t}{=k}_{H}t^{0.5}$
Korsmeyer–Peppas	$\log\left(\frac{m_{t}}{m_{\infty }}\right){=\mathrm{logk}}_{K-P}+\mathrm{nlogt}$
Hixon–Crowell	$m_{0}{}^{1/3}{-m}_{\mathrm{rtnd}}{}^{1/3}{=k}_{H-C}t$

where m_t_—the amount of the drug released during time t; m_b_—the amount of the drug in the solution before the release, usually 0; k_0_—the zero-order release rate constant; m_0_—the amount of the drug in the formulation before the dissolution; k_1_—the first-order release rate constant; k_2_—the second-order release rate constant; k_H_—the Higuchi rate constant; m_∞_—the amount of the drug released after an infinitive time, k_K-P_—the Korsmeyer–Peppas rate constant, n—the parameter indicative of the drug release mechanism; m_rtnd_—the amount of the drug retained in the formulation after time t; k_H-C_—the Hixon-Crowell rate constant.

## Supplementary Materials

The following are available online, Figure S1: The microscopic images of all formulations studied, Figure S2: The DSC thermograms of F1 (green line), F6 (red line) and physical mixture of F6 (blue line), Table S1. The values of Tukey’s HSD test obtained for the comparison of diameter wet beads (HSD = 0.146). Table S2: The values of Tukey’s HSD test obtained for the comparison of diameter dry beads (HSD = 0.127), Table S3: The mean diameter of wet and dry beads of formulations F1–F10.
