# Supplementary material for: Bipolymeric Pectin Millibeads Doped with Functional Polymers as Matrices for the Controlled and Targeted Release of Mesalazine
Source: Molecules. 2020 Dec 3;25(23):5711. doi: 10.3390/molecules25235711 (PMC7731135; doi:10.3390/molecules25235711)
Supplement: Supplementary file 1 [file molecules-25-05711-s001.pdf]

Supplementary materials

Table S1. The values of Tukey's HSD test obtained for the comparison of diameter wet beads (HSD=0.146).

	F1	F2	F3	F4	F5	F6	F7	F8	F9	F10
F1	0.000	0.190	0.160	0.150	0.150	0.280	0.340	0.350	0.360	0.340
F2		0.000	0.030	0.040	0.040	0.090	0.150	0.160	0.170	0.150
F3			0.000	0.010	0.010	0.120	0.180	0.190	0.200	0.180
F4				0.000	0.000	0.130	0.190	0.200	0.210	0.190
F5					0.000	0.130	0.190	0.200	0.210	0.190
F6						0.000	0.060	0.070	0.080	0.060
F7							0.000	0.010	0.020	0.000
F8								0.000	0.010	0.010
F9									0.000	0.020
F10										0.000

Table S2. The values of Tukey's HSD test obtained for the comparison of diameter dry beads (HSD=0.127).

	F1	F2	F3	F4	F5	F6	F7	F8	F9	F10
F1	0	0.013	0.003	0.054	0.024	0.174	0.164	0.164	0.179	0.120
F2		0.000	0.010	0.067	0.037	0.187	0.177	0.177	0.192	0.133
F3			0.000	0.058	0.027	0.178	0.167	0.167	0.182	0.124
F4				0.000	0.030	0.120	0.110	0.110	0.124	0.066
F5					0.000	0.150	0.140	0.140	0.155	0.097
F6						0.000	0.010	0.010	0.004	0.054
F7							0.000	0.000	0.015	0.044
F8								0.000	0.015	0.044
F9									0.000	0.058
F10										0.000

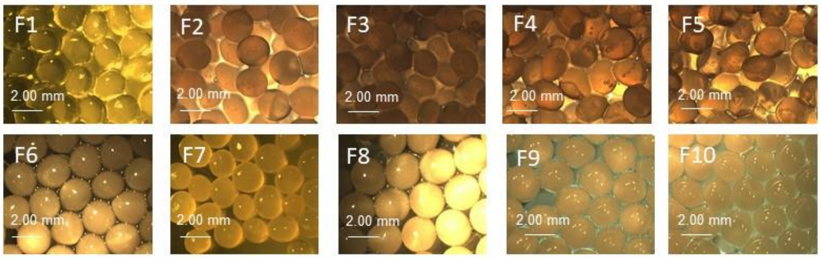

Figure S1. The microscopic images of all formulations studied.

Table S3. The mean diameter of wet and dry beads of formulations F1–F10

	F1	F2	F3	F4	F5	F6	F7	F8	F9	F10
Mean diameter of wet beads [mm]	1.47 ±0.21	1.66 ±0.15	1.63 ±0.15	1.62 ±0.18	1.62 ±0.20	1.75 ±20	1.81 ±0.29	1.82 ±0.20	1.83 ±0.30	1.81 ±0.30
Mean diameter of dry beads [mm]	0.85 ±0.18	0.82 ±0.14	0.82 ±0.14	0.86 ±0.13	0.83 ±0.14	0.99 ±0.22	1.03 ±0.20	0.98 ±0.22	0.98 ±0.18	0.92 ±0.15

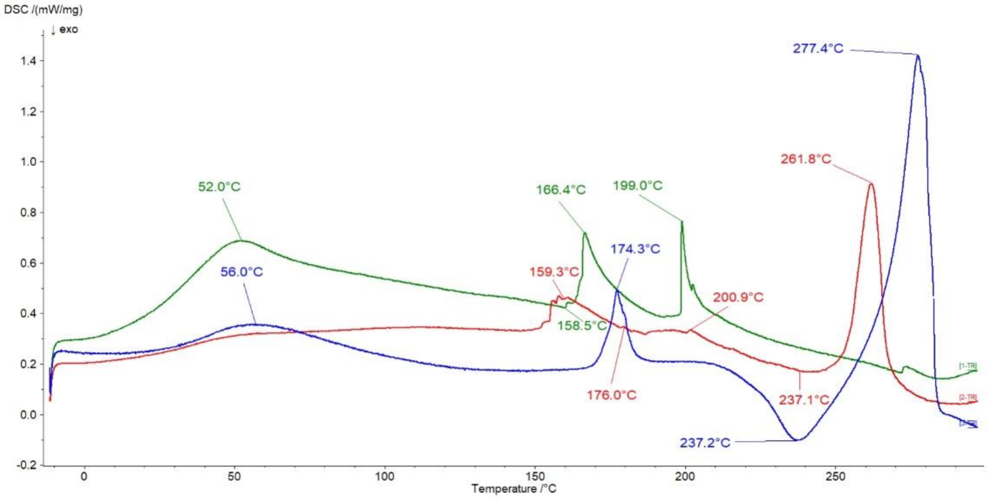

Figure S2. The DSC thermograms of F1 (green line), F6 (red line) and physical mixture of F6 (blue line).
